# Use of Recommended Search Strategies in Systematic Reviews and the Impact of Librarian Involvement: A Cross-Sectional Survey of Recent Authors

**DOI:** 10.1371/journal.pone.0125931

**Published:** 2015-05-04

**Authors:** Jonathan B. Koffel

**Affiliations:** Bio-Medical Library, University of Minnesota, Minneapolis, Minnesota, United States of America; McGill University, CANADA

## Abstract

**Background:**

Previous research looking at published systematic reviews has shown that their search strategies are often suboptimal and that librarian involvement, though recommended, is low. Confidence in the results, however, is limited due to poor reporting of search strategies the published articles.

**Objectives:**

To more accurately measure the use of recommended search methods in systematic reviews, the levels of librarian involvement, and whether librarian involvement predicts the use of recommended methods.

**Methods:**

A survey was sent to all authors of English-language systematic reviews indexed in the Database of Abstracts of Reviews of Effects (DARE) from January 2012 through January 2014. The survey asked about their use of search methods recommended by the Institute of Medicine, Cochrane Collaboration, and the Agency for Healthcare Research and Quality and if and how a librarian was involved in the systematic review. Rates of use of recommended methods and librarian involvement were summarized. The impact of librarian involvement on use of recommended methods was examined using a multivariate logistic regression.

**Results:**

1560 authors completed the survey. Use of recommended search methods ranged widely from 98% for use of keywords to 9% for registration in PROSPERO and were generally higher than in previous studies. 51% of studies involved a librarian, but only 64% acknowledge their assistance. Librarian involvement was significantly associated with the use of 65% of recommended search methods after controlling for other potential predictors. Odds ratios ranged from 1.36 (95% CI 1.06 to 1.75) for including multiple languages to 3.07 (95% CI 2.06 to 4.58) for using controlled vocabulary.

**Conclusions:**

Use of recommended search strategies is higher than previously reported, but many methods are still under-utilized. Librarian involvement predicts the use of most methods, but their involvement is under-reported within the published article.

## Introduction

Systematic reviews are considered to provide the best evidence for health care decisions by gathering, appraising and synthesizing all available literature around a question in a systematic and reproducible manner [1, 2]. The foundation of any systematic review is a comprehensive and systematic search that identifies all eligible studies on the topic and utilizes specialized search tools and methods [3]. Although several prominent organizations have created guides on how to conduct and report effective searches [2, 4–7], many published systematic reviews have been found to contain errors in the design and conduct of the searches that could affect their quality [8–10]. In addition, many reviews have incomplete reporting of the search strategy in the published article [9, 11–15]. Due to the poor search reporting, it is very difficult to know if searches are being *conducted* poorly or just *reported* poorly.

One key recommendation for how to improve search quality has been to include a librarian or search specialist (hereafter librarian) when designing and conducting the literature search [2, 4, 5]. Librarians have regularly advocated for their increasing involvement in systematic reviews [10, 16–19] and accumulating evidence supports this advocacy by showing that search quality and reproducibility may be improved when a librarian is involved [9, 20]. Despite these promising findings, existing studies have been limited to examining data available in the published articles and thus may be biased by the poor reporting of search strategies in published articles. In addition, many articles do not provide enough information to determine who conducted the search and their credentials [9, 15, 21], so it is unclear what the true rate of librarian involvement may be. A more accurate measure of librarian involvement is crucial for continued progress in determining librarian impact.

This study will make important contributions to the literature on search strategies and librarian involvement in systematic reviews in two ways. First, it will directly survey recent systematic review authors to determine their use of recommended search strategies and compare these to rates in previous studies. This will allow a larger and more generalizable sample than in previous studies and circumvents the issue of poor reporting, allowing us to determine if searches are being *conducted* poorly or just *reported* poorly. Second, it will survey authors on the involvement of a librarian in the review and their role, providing a significantly more accurate and complete estimate than in previous studies. The impact of librarian involvement on the use of recommended search strategies and methods will be examined with the hypothesis that their involvement will be associated with higher use of these strategies.

## Methods

### Citation Identification

Systematic reviews published in January, 2012 through January, 2014 were identified in the Database of Abstracts of Reviews of Effects (DARE), which conducts highly-sensitive weekly searches of a wide range of sources to identify new systematic reviews [22]. A two-year publication window was chosen to balance the need for a representative and robust sample with authors’ ability to recall the specifics of their search strategy. Systematic reviews from the Cochrane Database of Systematic Reviews were excluded since they follow specific guidelines, represent a subset of all published systematic reviews and are not representative of those in general or specialty journals [12, 13, 23]. DARE was selected since existing hedges and filters for Embase/MEDLINE have sub-optimal precision [24] and thus are inefficient for identifying large numbers of systematic reviews.

The citations from DARE were enriched with additional citation and abstract information from the Scopus/Embase [25] and Web of Science [26] databases by matching digital object identifiers (DOIs) and PubMed ID (PMID) numbers. The ISI Impact Factor for each journal was extracted from the 2013 Journal Citation Reports [27] and converted to its relative quartile rank within its subject category. Since impact factor ranges can vary between disciplines [28], this normalization allowed journals’ impact factors to be more directly compared.

The retrieved citation data was cleaned and elements such the corresponding author’s country and email address were extracted from the address field and placed in unique fields. Only English language articles where an email address for the corresponding author could be identified were included in the study.

### Survey

An online survey was designed to gather information on the authors’ background with systematic reviews and searching, search methods in a specific review, and experiences collaborating with librarians (S1 Text). Items on the conduct of the search were gathered from existing lists of best practices for systematic review searches and reporting [2, 4, 5, 12, 29]. Items on the systematic review process and author background were modified and expanded from those used in a previous study [30]. Additional items were developed based on author knowledge and review of previous trends and findings. The survey was pilot-tested and reviewed by local systematic review authors, systematic review experts and search experts for content and validity.

In order to ensure that each corresponding author was only contacted once and was only associated with a single article, a copy of the list of candidate articles was generated and duplicate email addresses were removed. When appropriate, the most recent article from each author was chosen. Each author was sent an e-mail invitation to participate in the study and a unique survey link. The invitation included a description of the current study, the title of the specific article associated with the author in the data set and instructions to answer the questions about the search strategy used in that specific article. The article title was stated again in the survey introduction. Due to the potentially sensitive nature of the survey, all responses were anonymous.

### Analyses

In order to determine the proportion of systematic reviews using recommended search methods, the frequency of each method’s use was computed. If a respondent reported that they were “unclear” if a search method was used, it was coded as “no” in order to provide a more conservative estimate and a sensitivity analysis was conducted.

In order to determine the impact of librarian involvement on use of recommended search methods, the frequency of librarian involvement and nature of that involvement was calculated. In addition, information was gathered from authors on variables beyond librarian involvement that have a demonstrated or plausible effect on systematic review quality, and thus potentially on the use of recommended search methods. These include specialized knowledge in or training on systematic review methods [31, 32], publishing in a higher impact journal [32, 33], previous experience writing systematic reviews [34], use of a reporting guidelines [35, 36], years of professional practice, time taken to write the review, year of publication and self-reported confidence in systematic review methodology and the review topic area.

A multivariate logistic regression was conducted to determine the impact of librarian involvement on the use of each recommended search method, controlling for variables reviewed above. These variables were entered in a single step as a covariate block, with librarian involvement entered as the second step. Odds ratios, confidence intervals and significance values for the association between librarian involvement and search method use were calculated.

Differences in demographic variables for included and excluded articles were examined using a χ^2^test.

All analyses were conducted using SPSS (version 22) and a significance level of α =. 05 was used.

Missing data was not imputed due to the nature of the data and responses were included if the respondent had answered at least two questions. Frequencies were chosen over raw counts due to varying response rates to each question and for ease of interpretation.

## Results

### Article and Respondent Characteristics

**Fig 1:**
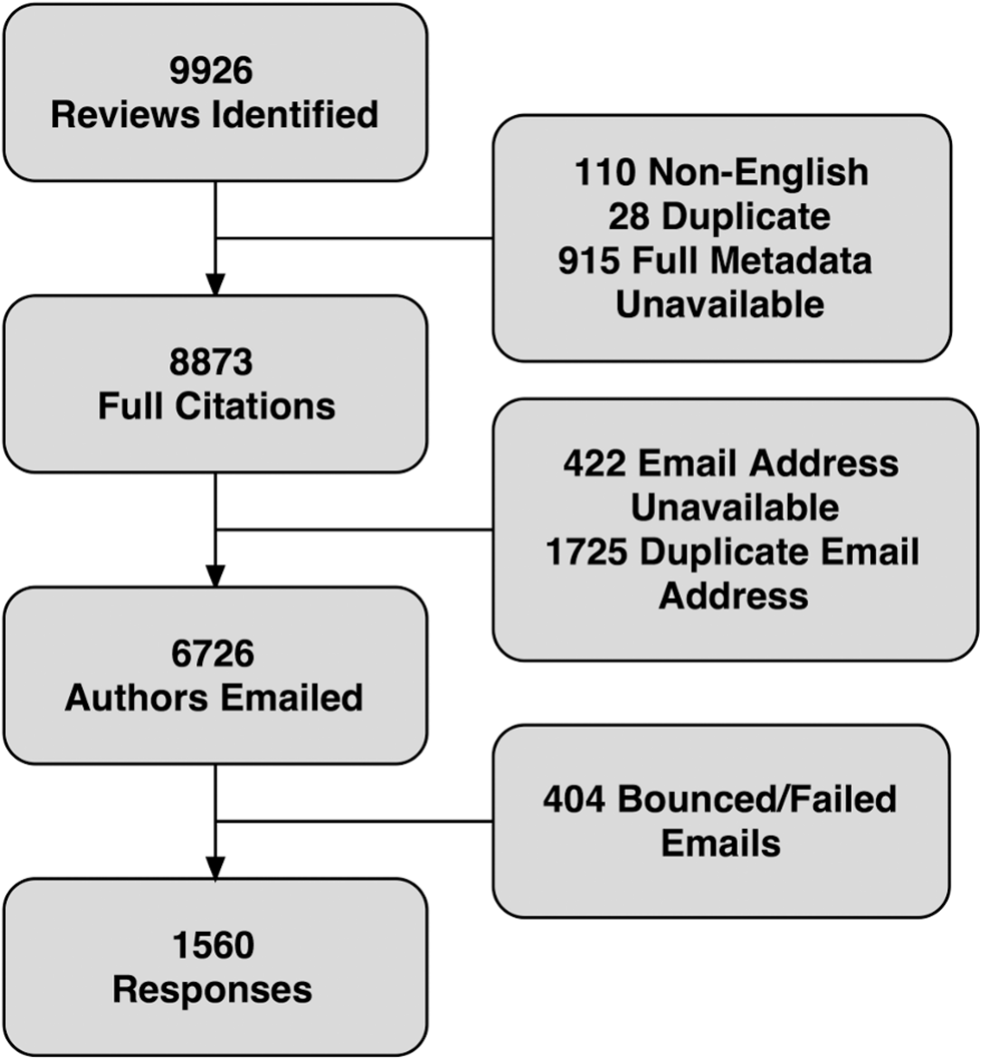
Identification of Included Articles.

A search was conducted in the DARE database on February 2^nd^, 2014 and citations for 9,926 articles were retrieved (Fig 1). Expanded citation information could be extracted from the Scopus (which includes Embase) and Web of Science databases for 8,873 of these and a list of 6,726 unique author emails was generated. The survey was successfully sent to 6,322 authors and fully or partially completed by 1,560 of these (25% response rate).

Characteristics of the full list of articles (n = 8873) and those articles where the author opened the survey (n = 1638) are described in Table 1. Due to the anonymity of the responses, for this analysis it was only possible to identify articles where the author had opened the survey, not those where the author had fully or partially completed it. This resulted in a small number (78 articles, 5%) being incorrectly counted as included.

**Table 1:** Article Characteristics.

	All Articles *(n = 8873)*	Non-Respondents *(n = 7235)*	Respondents *(n = 1638)*	
**Years % (n)**				p<.001
2012	40% (3523)	42% (3019)	31% (504)	
2013	57% (5032)	55% (3965)	65% (1067)	
2014	4% (318)	4% (251)	4% (67)	
**Country of Corresponding Author (top 5 by %) % (n)**				p<.001
China	28% (2522)	33% (2352)	10% (170)	
United States	17% (1470	16% (1147)	20% (323)	
United Kingdom	12% (1029)	11% (820)	13% (209)	
Canada	7% (646)	7% (509)	8% (137)	
Australia	6% (503)	5% (372)	8% (131)	
**Journal Rank by Quartile % (n) §**				p<.001
1^st^ Quartile	48% (4229)	46% (3355)	53% (874)	
2^nd^ Quartile	24% (2160)	24% (1749)	25% (411)	
3^rd^ Quartile	15% (1310)	16% (1141)	10% (169)	
4^th^ Quartile	6% (525)	6% (455)	4% (70)	
Missing	7% (649)	7% (535)	7% (114)	
**Number of Authors (median, IQR)**	5 (4–7)	5 (4–7)	5 (3–6)	p<.001
**Indexed as SR in Embase % (n)**	87% (6579)	87% (5417)	87% (1162)	p = 1.0
**SR terms in the Title/Abstract % (n)**	93% (8240)	93% (6736)	92% (1504)	p =. 07

**§** According to 2013 Journal Citation Reports.

All comparisons between respondent and non-respondent articles. SR = systematic review/meta-analysis, IQR = interquartile range.

Included articles differed significantly from those not included in terms of year (higher prevalence of 2013 articles), journal quartile (higher prevalence of first quartile articles), number of authors and the corresponding author’s country. While the top five countries of the corresponding authors are identical, a larger proportion of respondents came from English-speaking countries and fewer from China. The strong majority of articles had the words “systematic review” or “meta(-)analysis” in the title or abstract of the article (92%) and most (87%) were indexed as systematic reviews or meta-analyses by the Embase literature database.

Characteristics of the respondents’ background and on pre-defined covariates that will be included in the regression analysis are presented in Table 2. Authors tended towards being earlier in their careers with a median of 8 years of professional experience and 75% having 15 or fewer years of experience. While authors had completed a median of 3 previous reviews, there was wide variation (1–300) with 17% reporting having written 10 or more.

**Table 2:** Author and Study Design Characteristics.

Characteristic (n)	
**Author from Medical Discipline (1550)** *% (n)*	59% (907)
**Years of Experience (1516)** (*Median*, *IQR)*	8 (4–15)
**Number of Completed SRs (1537)** *(Median*, *IQR)*	3 (2–6)
**Time to Complete SR (1526)** *Months (Median*, *IQR)*	8 (5–12)
**Formal SR Training (1542)** *% (n)*	52% (808)
Local university course or workshop	76% (566)
Cochrane Collaboration workshop	18% (132)
Joanna-Briggs Institute	2% (14)
Local library or other	2% (18)
**SR Methodology Knowledge (1526)** (*Mean*, *SD)* §	3.62 (.84)
**SR Topic Knowledge (1536)** *(Mean*, *SD)* §	2.92 (1.28)
**Librarian Involved in the SR (1456)** *% (n)*	51% (739)
Design and execute all or part of the search strategy	55% (408)
Peer-review search strategy	38% (287)
Write up the search strategy for the article	19% (142)
Write or edit portions of the paper (other than the description of the search strategy)	9% (64)
Assist with statistical analyses	8% (63)
Consulted Only	8% (60)
**Librarian Acknowledgment (753)** *% (n)* ‡	64% (485)
Co-authorship	26% (195)
Referenced in the text	8% (63)
Referenced in the acknowledgments	33% (251)
Not formally recognized	36% (268)
**Followed Reporting Guideline (1441)** *% (n)*	88% (1273)
PRISMA	67% (844)
MOOSE	6% (71)
Cochrane Handbook	20% (257)
Other/Combination	7% (95)

IQR = interquartile range, SD = standard deviation, SR = systematic review/meta-analysis.

**§** Self-assessed with Likert Scale (1–5, novice to expert).

‡ Among those who involved a librarian.

Half (52%) of authors reported having received formal training in the conduct of systematic reviews. In the majority of cases, this training was through a university course rather than a formal workshop from the Cochrane Collaboration or other outside group.

Half (51%) of authors reported that a librarian was involved in the review in some way. Librarian contributions were typically recognized through authorship (26%) or in the acknowledgments (33%). In 36% of cases, including 25% of those where the librarian created or executed the search, their contributions were not recognized in the publication.

### Use of Recommended Search Methods

Table 3 reports the percentage of authors who reported the use of each of the recommended search strategies in this study and comparable rates from previous studies.

**Table 3:** Use of Recommended Methods and Librarian Impact.

		Use of Recommended Methods % (n)		Impact of Librarian Involvement OR (95% CI)	
**Recommended Search Strategy/Method [2, 4, 5]**		**Current Study *N = 1476***	**Previous Studies [9–13, 20, 33, 37–50]**	**Unadjusted Odds Ratio *N = 1456***	**Adjusted Odds Ratio *N = 1271***
**Traditional Methods**					
1	> 2 databases searched	96% (1421)	32–95%	3.58 (1.86–6.88) ***	3.07 (1.50–6.27) **
2		98% (1439)	22–73%	.78 (.40–1.51)	.60 (.28–1.30)
3	Controlled vocabulary (MeSH) used	88% (1292)	18–26%	3.64 (2.56–5.19) ***	3.07 (2.06–4.58) ***
4	Synonyms used	91% (1347)	63–94%	1.81 (1.24–2.63) **	1.72 (1.12–2.66) **
5	Boolean logic used	97% (1434)	6–50%	3.67 (1.73–7.76) ***	2.89 (1.07–7.77) *
6	Search adjusted for each database	70% (1032)	none	1.66 (1.32–2.08) ***	1.49 (1.15–1.92) **
7	Multiple languages searched	64% (945)	19–60%	1.41 (1.13–1.74) **	1.36 (1.06–1.75) **
**Extended Methods**					
8	Grey literature searched	53% (776)	8–65%	1.68 (1.36–2.07) ***	1.66 (1.31–2.09) ***
9	Journals handsearched	40% (584)	6–84%	1.38 (1.12–1.71) **	1.36 (1.07–1.73) **
10	Clinical trial registries searched	47% (694)	32%	1.21 (.99–1.49)	1.09 (.86–1.38)
11	Citation indices searched	57% (840)	2–28%	1.21 (.98–1.49)	1.23 (.97–1.55)
12	References in articles reviewed	97% (1425)	48–78%	.80 (.46–1.42)	.73 (.38–1.37)
13	Prominent authors contacted	51% (755)	10–28%	1.41 (1.14–1.73) ***	1.26 (.99–1.58)
**Search Process/Reporting**					
14	Search results updated during process	86%(1276)	72%	.933 (.69–1.26)	.76 (.54–1.07)
15	Search strategy peer-reviewed	60% (884)	1%	1.83 (1.48–2.26) ***	1.92 (1.51–2.44) ***
16	SR registered in PROSPERO	9% (136)	none	1.77 (1.23–2.56) **	1.63 (1.07–2.48) *
17	Full strategy provided	71% (1045)	18–43%	2.11 (1.67–2.66) ***	1.88 (1.44–2.45) ***

Significant associations in bold.

* p<.05

** p<.01

***p<.001.

CI = confidence interval. SR = systematic review/meta-analysis. Covariates of adjusted odds ratio: formal training in systematic review methodology, journal impact factor quartile, number of previous systematic reviews, use of a reporting guidelines, years of professional practice, time taken to write the review, year of publication and self-reported confidence in systematic review methodology and the topic area of the systematic review.

Use of recommended traditional search methods (items 1–7) was generally high and the majority ranged from 88%-98%. Lower rates were reported for searching in multiple languages (64%) and adjusting the strategy for each literature database (70%). In all cases, rates were higher than in prior studies that examined the published literature only. In some cases, such as use of controlled vocabulary, rates in the current study were markedly higher.

Use of extended search methods (items 8–13) were generally much lower than for traditional methods and the majority ranged from 40%-57%. Nearly all authors (97%), however, reported reviewing the references of included articles. As with traditional search methods, rates were often considerably higher than in comparable studies. Handsearching and use of grey literature were higher in some prior studies, though this may be due to the specific disciplines or topics studied in these articles.

Use of recommended methods for search management and reporting (items 14–17) were mixed. While 60%-80% of authors reported using most of the methods, only 9% reported registering their study in the PROSPERO systematic review register. As with previous methods, the rates in the current study were considerably higher than in previously published studies.

### Librarian Impact on Use of Recommended Search Methods

The association between librarian involvement and the use of each search method was investigated using a logistic regression and reported in Table 3, controlling for variables that have a demonstrated or plausible effect on systematic review quality.

Librarian involvement was found to be significantly associated with 12 out of 17 search items (71%) in a univariate (unadjusted) regression and 11 out of 17 items (65%) in a multivariate (adjusted) regression controlling for covariates. Adjusted odds ratios ranged from 1.36 to 3.07. Associations were strongest and most prevalent with traditional searching items (items 1–7), though there were also associations with items related to extended search methods (items 8–13) and the management and reporting of the review (items 14–17).

A sensitivity analysis was performed to determine the effect of coding responses of “unclear” for search elements as “yes” rather than “no” (S2 Text). All existing associations remained significant and, in the majority of cases, became larger. In addition, in the sensitivity analysis librarian involvement was found to significantly predict use of a citation index.

### Impact of Guideline Use and Systematic Review Training and Knowledge

As a post-hoc analysis, the association between each of the co-variates in the regression analysis (listed below Table 3) and the use of recommended strategies was investigated. For most of the variables examined, the associations were weak or non-significant. Three variables, however, did show a pattern of significant associations with the use of recommended search methods (S3 Text). Use of a reporting guideline was associated with use of 10 out of 17 methods (59%) with adjusted odds ratios ranging from 2.01–6.69. Formal training in systematic review methodology was associated with use of 6 out of 17 methods (35%) with adjusted odds ratios ranging from 1.32–1.96. Finally, an increase (one point on a five point scale) in self-reported knowledge of systematic review methodology was associated with the use of 8 out of 17 methods (47%) with adjusted odds ratios ranging from 1.18–2.20.

## Discussion

This study provides the largest examination to date of search strategies and librarian involvement in systematic reviews and is the first to survey authors across disciplines directly rather than relying on published strategies. By surveying authors directly, it was able to control for the generally poor reporting of systematic reviews and generate a more accurate estimate of the use of recommended search methods and the impact and levels of librarian involvement.

Use of recommended search strategies was generally high (see Table 3), and was equivalent or higher than reported in comparable studies examining article-reported rates in most cases. For some items, such as use of controlled vocabulary, it was considerably higher. In other cases, such as inclusion of a full search strategy, it was difficult to make an accurate comparison since definitions differ widely [14]. Major et al. [30] provide the only known study where author-reported search strategies in dental systematic reviews were compared with article-reported search strategies and they found only fair levels of agreement between the two sources with author-reported rates higher in all cases. By surveying authors directly, the current study may have generated a more accurate or complete measure of the use of recommended search methods. Although the findings regarding the frequency of use of recommended search methods are encouraging, certain recommended methods are still underused. Specifically, authors could improve their searches by adapting the strategy to each source, including multiple languages, searching for unpublished or grey literature, updating and peer-reviewing their searches and ensuring that enough information is provided in the article to allow the search strategy to be appraised and reproduced.

In this study, half of all authors reported involving a librarian or search specialist in the systematic review, often as the designer of the search strategy. This is significantly higher than in other studies that found at most a quarter of reviews involved a librarian [9–11, 20, 21, 51]. This difference is likely due to many authors not reporting librarian contributions in the published article, as was the case in a third of cases in this study. Librarian involvement was strongly associated with use of the majority of recommended search methods, even after controlling for potential confounding variables. This parallels previous studies that found an association between stated librarian involvement and search quality in published reviews. As part of a larger examination of search strategies in systematic reviews of adverse effects, Golder et al. [9] found that searches conducted by information professionals were more likely to be reproducible, include more search terms and search more databases. While the current study did not look at number of search terms used, librarians were significantly associated with searching two or more databases and with including the full search strategy. More recently, Rethlefsen et al. [20] found that librarian involvement predicted higher-quality and more reproducible searches, with a stronger effect for librarian co-authorship, in systematic reviews from high-impact general medicine journals. While this study and the current one found many similar associations, they found a negative association with handsearching in contrast to the positive association in this study. The differences between these studies and the current one are minor and most likely attributable to differences in the journals and topics included. The results show that systematic review authors should ensure that they are following best practices for conducting and reporting searches in their reviews and consider working with librarians as a method to improve the quality of their searches.

While this study was focused on the impact of librarian involvement, the post-hoc analysis found that use of reporting guidelines, formal training in systematic review methodology, and increased self-reported knowledge of systematic review methodology were also associated with the use of many recommended methods. This is exciting since, along with librarian involvement, these are modifiable factors and suggest ways to increase systematic review quality on a larger scale. Additional research, however, is necessary to better understand the associations and mechanisms. For instance, this study only asked about the use of reporting guidelines, which focus on what information needs to be included in the published paper and thus are unlikely to directly affect the methods used. It is probable that authors who follow reporting guidelines are more likely to follow guidelines on the conduct of the review, but this has yet to be established. Similarly, more research is needed on what training methods or self-paced information resources are associated with gains in systematic review and search strategy quality. As the number of systematic reviews grows, identifying interventions such as these that might improve quality will become increasingly important.

This study had several limitations. First and foremost, it is based on self-report, potentially several years after the event, and it is possible authors did not know what each recommended method entailed or had forgotten the details. For example, two-thirds of authors reported including a full search strategy in the published article, but examinations of published articles have found much lower inclusion rates ranging from 9–40%, indicating a disconnect between what authors believe they are doing and what is actually happening [14, 20]. For convenience in obtaining a large sample, systematic reviews were drawn from a pre-identified list of systematic reviews (DARE). While DARE aims to be as comprehensive as possible [22], it is likely that some systematic reviews were missed. In addition, DARE is focused on systematic reviews that address health-related topics, so the results of this study may not generalize to systematic reviews in the social sciences or other disciplines examining non-health questions. Only English-language articles were included to match the English-language survey, which could have resulted in a bias towards methods used in English-speaking countries. Systematic reviews published by the Cochrane Collaboration were excluded from this study since they are required to follow the guidelines of the Cochrane Collaboration [2], which include recommending the use of a Trial Search Coordinator or similar expert, and have been shown to have different quality and reporting of search strategies than non-Cochrane reviews [12, 13, 23]. Cochrane reviews represent a subset of the total number of systematic reviews published annually, estimated at 20% by Moher et al. in 2004 [13] and currently at 13% (869 new or updated Cochrane reviews published in 2014 [52] and 6589 non-Cochrane reviews published in 2014 indexed in DARE [22]). While excluding them means that the results cannot be generalized to Cochrane reviews, the results are generalizable to the majority of systematic reviews and are less likely to be biased by the required methods of any single publisher. Nevertheless, it would be interesting for future research to look at Cochrane reviews and see if their more proscribed methods decrease the association between librarian involvement and use of recommended methods. Finally, the response rate was low (25%) and it is possible that authors were more likely to respond, and thus bias the results, if they had collaborated with a librarian or felt they had conducted a high-quality search. Based on the demographics in Table 1, however, there are few meaningful differences between responders and non-responders. The main exception was a lower response rate from Chinese authors, but this may be expected for an English-language survey and it is unclear that Chinese authors differ from those from other countries on study variables. A more targeted survey with higher levels of participation would be needed to determine if a response bias was present.

A first target for future research would be a multi-methods approach that directly compares author-reported and article-reported search strategies. This would allow us to directly measure reporting deficits and improve on the indirect comparisons performed in this study. Research should also investigate whether authors understand the recommended search methods and reporting requirements, such as what constitutes including a full, reproducible strategy in the article. It is possible that authors believe they are following best practices when they are not. Finally, this and other studies have found that librarian involvement is associated with higher-quality searches, but it is unclear if this is due to correlation or causation. Future research could seek to more directly measure and quantify the specific impact of the librarian on the search process and articles retrieved.

## Conclusion

This study has shown that author-reported searching in systematic reviews is better than previously reported, but still sub-optimal. In particular, use of methods such as grey literature searching, adjusting terms and methods for each literature database and reporting of reproducible search strategies need to be improved. Librarian involvement was strongly associated with the use of many recommended search methods and could improve the quality of the review, contributing to the replicability and robustness of meta-analytic findings. It is important to note that librarian involvement uniquely contributed to search strategies after controlling for potential confounders. Despite the demonstrated positive impact of librarian involvement on the use of recommended search strategies and the relative ease with which this variable can be modified, librarian involvement is still low and their contributions need to be better acknowledged in the published article. A comprehensive and reproducible search strategy is core to any systematic review, and librarians represent important collaborators in the endeavor to produce high quality reviews.

## Supporting Information

S1 Data Article Characteristics—Cleaned Data. Contains information about each of the eligible articles and its corresponding author for this study. This includes both authors that completed the survey and those that did not. Data has been cleaned for consistency and to address data errors. Comma-delimited file. Field definitions and coding information are inserted below the data.
(CSV)

S2 Data Survey Responses—Cleaned Data. Contains the survey responses relevant to this study from each participant. Data has been cleaned and recoded for consistency and to address data errors. Comma-delimited file. Field definitions and coding information are inserted below the data.
(CSV)

S1 Text Survey Questions. (PDF)

S2 Text Sensitivity Analysis. (DOCX)

S3 Text Covariate Associations. (DOCX)
