# Supplementary material for: Use of Recommended Search Strategies in Systematic Reviews and the Impact of Librarian Involvement: A Cross-Sectional Survey of Recent Authors
Source: PLoS One. 2015 May 4;10(5):e0125931. doi: 10.1371/journal.pone.0125931 (PMC4418838; doi:10.1371/journal.pone.0125931)
Supplement: S1 Text — (PDF) [file pone.0125931.s003.pdf]

What is your primary discipline?

- ❍ Medicine (please indicate discipline/specialty below, if relevant) ____________________
- ❍ Nursing (please indicate discipline/specialty below, if relevant) ____________________
- ❍ Pharmacy (please indicate discipline/specialty below, if relevant) ____________________
- ❍ Dentistry (please indicate discipline/specialty below, if relevant) ____________________
- ❍ Public Health (please indicate discipline/specialty below, if relevant) ____________________
- ❍ Veterinary Medicine (please indicate discipline/specialty below, if relevant) ____________________
- ❍ Other ____________________

Which of the following best describes your clinical/research balance?

- ❍ Primarily clinical
- ❍ Primarily research
- ❍ Even balance of clinical and research
- ❍ Other ____________________

Were you the primary person responsible for the design and writing of this systematic review?

- ❍ Yes
- ❍ No
- ❍ Other ____________________

How many years has it been since you received your main professional degree? Please use numbers only

How many systematic reviews/meta-analyses have you completed or been a co-author on? Please use numbers only

How many systematic reviews have you begun, but not yet completed? Please use numbers only

Have you received any formal training in systematic review methodology prior to conducting your review?

- ❍ Yes (please indicate where) ____________________
- ❍ No

How knowledgeable do you consider yourself about systematic review methodology?

- ❍ Novice
- ❍
- ❍
- ❍
- ❍ Expert

How knowledgeable were you about the systematic review topic before beginning the review?

- ❍ Novice
- ❍
- ❍
- ❍
- ❍ Expert

How many months did it take to complete the systematic review? Please count from when you began work on the protocol or methodology to when the manuscript was first submitted to a journal. Please use numbers only.

Did you combine the results from multiple studies together and calculate a combined effect size? In other words, did you perform a meta-analysis?

- ❍ Yes
- ❍ No
- ❍ Unsure ____________________

Throughout this survey: "Search strategy" refers to the entire process you took to find studies to include in your systematic review. For instance, determining which article databases to search in and what terms to use, whether to hand-search specific journals, and whether to search clinical trial registries for unpublished studies. "Literature search" refers to searches in article databases such as PubMed/Medline, Embase, CENTRAL or Web of Science.

Who designed the search strategy for this systematic review?

- ❍ I designed/co-designed the search strategy
- ❍ A co-author (colleague or subject expert)
- ❍ A co-author (librarian or other search expert)
- ❍ A colleague or subject expert (not a co-author)
- ❍ A librarian or other search expert (not a co-author)
- ❍ Other ____________________

Where did you develop your literature searching skills? (select all that apply)

- ❑ Self-taught
- ❑ Training during professional education/residency
- ❑ Workshop/class on searching at my institution taught by a librarian or search expert
- ❑ Workshop/class on searching at my institution taught by someone other than a librarian or search expert
- ❑ Outside workshop or continuing education course (please specify) ____________________
- ❑ Other ____________________

What is your overall confidence in your literature searching skills?

- ❍ Not Confident
- ❍
- ❍
- ❍
- ❍ Very Confident

What is your overall confidence that your search strategy identified all relevant literature on your topic?

- ❍ Not Confident
- ❍
- ❍
- ❍
- ❍ Very Confident

Did you compare your search strategy to those conducted in similar systematic reviews?

- ❍ Yes
- ❍ No
- ❍ There were no similar systematic reviews
- ❍ Other ____________________

In what ways, if any, do you feel your search strategy could have been strengthened? (select all that apply)

- ❑ I feel that my search was strong and would not change anything
- ❑ Search additional databases/sources
- ❑ Broaden the literature search to retrieve more articles (increased sensitivity)
- ❑ Focus the literature search to retrieve more relevant articles (increased specificity)
- ❑ Search grey literature sources such as clinical trial registries or conference abstracts
- ❑ Work with a librarian or search expert
- ❑ Other ____________________

How many hours did it take to design and conduct your search strategy? Only count time spent actively designing, refining and executing your search strategy. Do not include time spent assessing articles for inclusion or time spent retrieving full-text for the articles.

- ❍ 0-5 hours
- ❍ 5-10 hours
- ❍ 10-20 hours
- ❍ 20-40 hours
- ❍ 40+ hours
- ❍ Other ____________________

How many articles did you expect your literature search to retrieve in order to be comprehensive? In other words, when searching in article databases as such PubMed/Medline, how many results did you expect to retrieve and have to screen for inclusion?

- ❍ 0 - 100
- ❍ 100 - 250
- ❍ 250 - 500
- ❍ 500 - 1,000
- ❍ 1,000 - 5,000
- ❍ 5,000 - 10,000
- ❍ Over 10,000

How many articles did you expect to meet your inclusion criteria and be included in your systematic review?

- ❍ 0 - 5
- ❍ 5 - 10
- ❍ 10 - 25
- ❍ 25 - 50
- ❍ 50 - 100
- ❍ Over 100

If you co-designed the search strategy, who was your co-designer? (please select all that apply)

- ❑ I did not have a co-designer
- ❑ A co-author (colleague or subject expert)
- ❑ A co-author (librarian or search expert)
- ❑ A colleague or subject expert (not a co-author)
- ❑ A librarian or search expert (not a co-author)
- ❑ Other _____________________

Which of the following were done in your review? If you did not design the search strategy, please answer to the best of your ability

	Yes	No	Unsure
Search two or more article databases (note: PubMed and Medline are the same database)	❍	❍	❍
Search using keywords (i.e., words searched in the title, abstract or full text, similar to Google)	❍	❍	❍
Search using Medical Subject Headings (MeSH terms) or other controlled vocabulary terms	❍	❍	❍
Use synonyms for keywords (e.g., heart attack, myocardial infarcation)	❍	❍	❍
Combine search terms with AND/OR/NOT (Boolean operators)	❍	❍	❍
Alter your literature search to take advantage of the unique capabilities (e.g., limits, controlled vocabulary) of each database	❍	❍	❍
Used limits to restrict your search to specific publication types, genders, ages, etc.	❍	❍	❍
Search for articles in more than one language	❍	❍	❍
Search clinical trial registries (e.g., clinicaltrials.gov)	❍	❍	❍
Search conference abstracts, white papers or other grey literature sources	❍	❍	❍
Search citation indices (Web of Science, Google Scholar, Scopus) to find articles that cited your included articles	❍	❍	❍
Review the reference lists of included studies to find additional articles	❍	❍	❍
Contact prominent authors to locate additional or unpublished articles	❍	❍	❍
Hand-search specific journals	❍	❍	❍
Re-run your search during the review process to find newly-published articles	❍	❍	❍
Have your search strategy peer-reviewed for quality and comprehensiveness	❍	❍	❍

Register your systematic review in PROSPERO (registry of in-progress and completed systematic reviews)	❍	❍	❍
Document your full literature search for at least one article database in a clear, reproducible manner in the article/appendix (example)	❍	❍	❍

What prevented you from searching two or more databases? (select all that apply)

- ❑ Limited time
- ❑ Did not think it was important/necessary
- ❑ Could not access other databases
- ❑ Did not know how to effectively search other databases
- ❑ Felt I found enough articles in the first database
- ❑ Felt all high quality articles were in the first database
- ❑ Actually, I did search two or more databases
- ❑ Other ____________________

What prevented you from searching using keywords? (select all that apply)

- ❑ Limited time
- ❑ Did not think it was important/necessary
- ❑ Did not know how to search using keywords
- ❑ Felt my search was comprehensive without using them
- ❑ Felt using keywords would retrieve too many results or irrelevant results
- ❑ Actually, I did search using keywords
- ❑ Other ____________________

What prevented you from searching using Medical Subject Headings (MeSH) or controlled vocabulary terms? (select all that apply)

- ❑ Limited time
- ❑ Did not think it was important/necessary
- ❑ Did not know how to search using MeSH or controlled vocabulary terms
- ❑ Felt my search was comprehensive without using them
- ❑ Felt using MeSH or controlled vocabulary terms would retrieve too many results or irrelevant results
- ❑ Actually, I did search using MeSH or controlled vocabulary terms
- ❑ Other ____________________

What prevented you from using synonyms for keywords? (select all that apply)

- ❑ Limited time
- ❑ Did not think it was important/necessary
- ❑ Did not use any keywords
- ❑ Felt my search was comprehensive without using them
- ❑ Could not identify any synonyms for my keywords
- ❑ Actually, I did use synonyms
- ❑ Other ____________________

What prevented you from using AND/OR/NOT (boolean operators) to combine search terms? (select all that apply)

- ❑ Limited time
- ❑ Did not think it was important/necessary
- ❑ Unsure how to use them to combine terms
- ❑ Retrieved too many results when I tried to use them
- ❑ Actually, I did use AND/OR/NOT
- ❑ Other ____________________

What prevented you from altering your literature search to take advantage of the unique capabilities (e.g., limits, controlled vocabulary) of each database? (select all that apply)

- ❑ Limited time
- ❑ Did not think it was important/necessary
- ❑ Did not realize different databases may need different strategies
- ❑ Did not know how to customize my search strategy for each database
- ❑ Actually, I did adapt my search strategy for each database
- ❑ Other ____________________

What prevented you from using limits to focus or refine your search? (select all that apply)

- ❑ Limited time
- ❑ Did not think it was important/necessary
- ❑ Did not know how to apply limits
- ❑ Available limits did not match what I needed
- ❑ Actually, I did use limits
- ❑ Other ____________________

What prevented you from searching for articles in more than one language? (select all that apply)

- ❑ Limited time
- ❑ Did not think it was important/necessary
- ❑ Did not have a way to read articles in another language
- ❑ Knew that all relevant literature was published in one language
- ❑ Actually, I did search for articles in multiple languages
- ❑ Other ____________________

What prevented you from searching in clinical trial registries? (select all that apply)

- ❑ Limited time
- ❑ Did not think it was important/necessary
- ❑ Did not know what clinical trial registries were available
- ❑ Did not know how to search effectively within the clinical trial registry
- ❑ Clinical trials were excluded from the systematic review
- ❑ Actually, I did search in a clinical trial registry
- ❑ Other ____________________

What prevented you from searching conference abstracts and other grey literature sources? (select all that apply)

- ❑ Limited time
- ❑ Did not think it was important/necessary
- ❑ Did not know where to find conference abstracts
- ❑ Did not know how to search effectively for conference abstracts
- ❑ Conference abstracts were excluded from the systematic review
- ❑ Actually, I did search for conference abstracts and other grey literature
- ❑ Other ____________________

What prevented you from searching citation indices (Web of Science, Google Scholar, Scopus) to find articles that cited your included articles? (select all that apply)

- ❑ Limited time
- ❑ Did not think it was important/necessary
- ❑ Did not know what citation indices were available
- ❑ Did not know how to search citation indices effectively
- ❑ Actually, I did search citation indices
- ❑ Other ____________________

What prevented you from reviewing the reference lists of included articles? (select all that apply)

- ❑ Limited time
- ❑ Did not think it was important/necessary
- ❑ Actually, I did review the reference lists of included articles
- ❑ Other ____________________

What prevented you from contacting prominent authors? (select all that apply)

- ❑ Limited time
- ❑ Did not think it was important/necessary
- ❑ Did not want to alert potential competitors that I was writing the review
- ❑ Could not identify relevant experts
- ❑ I am the most prominent researcher of this topic
- ❑ Actually, I did contact prominent authors
- ❑ Other ____________________

What prevented you from hand searching specific journals? (select all that apply)

- ❑ Limited time
- ❑ Did not think it was important/necessary
- ❑ Did not have access to copies of the relevant journals
- ❑ Could not identify journals to hand search
- ❑ Unsure how to hand search a journal
- ❑ Actually, I did hand search journals
- ❑ Other ____________________

What prevented you from re-running your literature search during the review process to find newly published articles? (select all that apply)

- ❑ Limited time
- ❑ Did not think it was important/necessary
- ❑ Could not replicate my search strategy
- ❑ Actually, I did re-run the search
- ❑ Other ____________________

What prevented you from having your search strategy peer-reviewed before conducting your search? (select all that apply)

- ❑ Limited time
- ❑ Did not think it was important/necessary
- ❑ Could not find a colleague or search expert to peer-review it
- ❑ Actually, I did have the search strategy peer-reviewed
- ❑ Other ____________________

What prevented you from registering your systematic review in PROSPERO? (select all that apply)

- ❑ Limited time
- ❑ Did not think it was important/necessary
- ❑ Process was too cumbersome
- ❑ Did not know about PROSPERO
- ❑ Actually, I did register my systematic review in PROSPERO
- ❑ Other ____________________

What prevented you from documenting your full literature search strategy for at least one article database in a clear, reproducible manner in the article/appendix? (select all that apply)

- ❑ Limited time
- ❑ Did not think it was important/necessary
- ❑ Did not have a record of my full search strategy
- ❑ Insufficient space in the article
- ❑ Editor asked that I remove it
- ❑ Peer-reviewer asked that I remove it
- ❑ Actually, I did document my full search strategy for at least one database in the article or appendix
- ❑ Other ____________________

You indicated that you did document your full literature search strategy for at least one article database in a clear, reproducible manner in the article/appendix.Where was the full search strategy reported?

- ❑ Article text
- ❑ Appendix
- ❑ Other ____________________

Did you (or your co-authors) work with a librarian, information specialist or other search expert as part of this systematic review?

- ❍ Yes
- ❍ No
- ❍ Unsure ____________________

What was the librarian or search expert's role? (please select all that apply)

- ❑ Design and execute all or part of the search strategy
- ❑ Peer-review search strategy
- ❑ Write up the search strategy for the article
- ❑ Write or edit portions of the paper (other than the description of the search strategy)
- ❑ Assist with statistical analyses
- ❑ Other ____________________

How were their contributions recognized in the paper? (please select all that apply)

- ❑ Co-authorship
- ❑ Referenced in the text
- ❑ Referenced in the acknowledgements
- ❑ Contributions were not explicitly recognized
- ❑ Other ____________________

Why did you choose to work with a librarian or information specialist? (please select all that apply)

- ❑ Worked with one on a previous systematic review
- ❑ Worked with one on a previous article (not a systematic review)
- ❑ Worked with one on clinical work
- ❑ Worked with one on a research project
- ❑ Heard about a library service supporting systematic reviews
- ❑ Recommended by a colleague
- ❑ Recommended by journal or systematic review guideline (please indicate journal or guideline) ____________________
- ❑ Just seemed like a good idea
- ❑ Unsure, my co-author made the decision
- ❑ Other ____________________

Why did you not work with a librarian or information specialist on this project? (please select all that apply)

- ❑ Unsure if we worked with one or not
- ❑ Limited time
- ❑ Did not think it was important/necessary
- ❑ Did not know they could help with systematic reviews
- ❑ Did not need help crafting the search strategy
- ❑ Concerns about potential cost
- ❑ Concerns about adding an additional co-author
- ❑ Did not know how to contact one
- ❑ Other ____________________

How much contact do you have with librarians or information specialists?

- ❍ Regularly work with them on research or patient care
- ❍ I have worked with them on several occasions
- ❍ I have worked with them rarely, but am aware who they are
- ❍ Do not work with them at all and am unsure how to contact them
- ❍ Other ____________________

What do you see as the benefits of working with a librarian or information specialist on a systematic review? (please select all that apply)

- ❑ Save time by distributing the work
- ❑ Increase the quality or comprehensiveness of the search or final article
- ❑ Increase the perceived methodological quality among editors and peer-reviewers
- ❑ Other ____________________

What do you see as the barriers to working with a librarian or information specialist on a systematic review? (please select all that apply)

- ❑ Requires extra time and planning
- ❑ May recommend searches that retrieve too many results
- ❑ They do not have the necessary subject expertise
- ❑ Potential cost
- ❑ May add additional authors to the paper
- ❑ Other ____________________

Did you follow a particular systematic review reporting guideline/standard when writing your review? Example: PRISMA, MOOSE, Cochrane Handbook, Institute of Medicine (IOM)

- ❍ Yes
- ❍ No

Which reporting guideline did you follow?

- ❍ PRISMA
- ❍ MOOSE
- ❍ Cochrane Handbook
- ❍ IOM (Institute of Medicine)
- ❍ Other ____________________

Why did you use this guideline? (please select all that apply)

- ❑ Required by the journal
- ❑ Recommended by the peer reviewers or journal editor
- ❑ Believed it would strengthen the article or increase its ability to be published
- ❑ Believed it was best practice
- ❑ Other ____________________

How well were you able to recall your experience of writing this systematic review?

- ❍ Not Well
- ❍
- ❍
- ❍
- ❍ Very Well

What could libraries or librarians do to help systematic review authors conduct their search strategy more easily or effectively?

Any final comments on how you conducted or designed the search strategy for this systematic review?
