# Supplementary material for: Use of Recommended Search Strategies in Systematic Reviews and the Impact of Librarian Involvement: A Cross-Sectional Survey of Recent Authors
Source: PLoS One. 2015 May 4;10(5):e0125931. doi: 10.1371/journal.pone.0125931 (PMC4418838; doi:10.1371/journal.pone.0125931)
Supplement: S2 Text — (DOCX) [file pone.0125931.s004.docx]

| **Supplementary File 2: Effects of recoding authors’ responses of “unclear” as “yes”** | | | | | | | |
| --- | --- | --- | --- | --- | --- | --- | --- |
|  | | **Use of Recommended Methods**  *N=1476, %(n)* | | |  | **Impact of Librarian Involvement**  *N=1271, Odds Ratio (95% CI)* | |
| Recommended Strategy/Method  ^2 4 5^ | | |  | Coded as “Yes” |  | Adjusted | Adjusted  Coded as “Yes” |
| **Traditional Methods** | | |  |  |  |  |  |
| 1 | > 2 databases searched | | 96% (1421) | 96% (1423) |  | **3.07 (1.50-6.27) **** | **3.28 (1.56-6.89) **** |
| 2 | Keywords used | | 98% (1439) | 98% (1447) |  | .60 (.28-1.30) | .81 (.34-1.95) |
| 3 | Controlled vocabulary (MeSH) used | | 88% (1292) | 90% (1325) |  | **3.07 (2.06-4.58) ***** | **3.43 (1.39-3.21) ***** |
| 4 | Synonyms used | | 91% (1347) | 94% (1382) |  | **1.72 (1.12-2.66) **** | **2.69 (1.58-2.98) ***** |
| 5 | Boolean logic used | | 97% (1434) | 98% (1448) |  | **2.89 (1.07-7.77) *** | **6.35 (1.36-29.72) *** |
| 6 | Search adjusted for each database | | 70% (1032) | 82% (1215) |  | **1.49 (1.15-1.92) **** | **1.87 (1.37-2.56) ***** |
| 7 | Multiple languages searched | | 64% (945) | 65% (964) |  | **1.36 (1.06-1.75) **** | **1.29 (1.45-2.38) *** |
| **Extended Methods** | | |  |  |  |  |  |
| 8 | Grey literature searched | | 53% (776) | 56% (820) |  | **1.66 (1.31-2.09) ***** | **1.72 (1.36-2.17) ***** |
| 9 | Journals handsearched | | 40% (584) | 42% (616) |  | **1.36 (1.07-1.73) **** | **1.35 (1.06-1.71) *** |
| 10 | Clinical trial registries searched | | 47% (694) | 50% (744) |  | 1.09 (.86-1.38) | 1.13 (.90-1.43) |
| 11 | Citation indices searched | | 57% (840) | 61% (902) |  | 1.23 (.97-1.55) | **1.39 (1.10-1.76) **** |
| 12 | References in articles reviewed | | 97% (1425) | 97% (1436) |  | .73 (.38-1.37) | .74 (.36-1.50) |
| 13 | Prominent authors contacted | | 51% (755) | 53% (784) |  | 1.26 (.99-1.58) | 1.24 (.98-1.57) |
| **Search Process/Reporting** | | |  |  |  |  |  |
| 14 | Search updated during process | | 86%(1276) | 87% (1288) |  | .76 (.54-1.07) | .76 (.54-1.08) |
| 15 | Search strategy peer-reviewed | | 60% (884) | 65% (959) |  | **1.92 (1.51-2.44) ***** | **2.04 (1.60-2.62) ***** |
| 16 | SR registered in PROSPERO | | 9% (136) | 13% (193) |  | **1.63 (1.07-2.48) *** | **1.89 (1.32-2.70) ***** |
| 17 | Full strategy provided | | 71% (1045) | 77% (1133) |  | **1.88 (1.44-2.45) ***** | **2.16 (1.62-2.88) ***** |
| Significant associations in bolded text. * p<.05, ** p<.01, ***p<.001. CI = confidence interval. SR = systematic review/meta-analysis. Covariates of adjusted odds ratio: formal training in systematic review methodology, journal impact factor quartile, number of previous systematic reviews, use of a reporting guidelines, years of professional practice, time taken to write the review, year of publication and self-reported confidence in systematic review methodology and the topic area of the systematic review. | | | | | | | |
