# Supplementary material for: Use of Recommended Search Strategies in Systematic Reviews and the Impact of Librarian Involvement: A Cross-Sectional Survey of Recent Authors
Source: PLoS One. 2015 May 4;10(5):e0125931. doi: 10.1371/journal.pone.0125931 (PMC4418838; doi:10.1371/journal.pone.0125931)
Supplement: S3 Text — (DOCX) [file pone.0125931.s005.docx]

| **Supplementary File 3: Associations of Selected Covariates with Use of Recommended Search Methods** | | | | |
| --- | --- | --- | --- | --- |
| Recommended Strategy/Method  ^2 4 5^ | | **Reporting Guideline Used**  *Odds Ratio (95% CI)* | **Training in Systematic Review Methods**  *Odds Ratio (95% CI)* | **Knowledge of Systematic Review Methods ‡**  *Odds Ratio (95% CI)* |
| **Traditional Methods** | |  |  |  |
| 1 | > 2 databases searched | **3.07 (1.52-6.20) **** | 1.36 (.72-2.57) | 1.12 (.75-.1.67) |
| 2 | Keywords used | .95 (.28-3.29) | 1.10 (.51-2.37) | 1.05 (.63-1.75) |
| 3 | Controlled vocabulary (MeSH) used | **2.41 (1.52-3.81) ***** | **1.96 (1.34-2.85) ***** | 1.21 (.96-1.53) |
| 4 | Synonyms used | 1.22 (.68-2.19) | 1.49 (.97-2.8) | **1.52 (1.17-1.99) **** |
| 5 | Boolean logic used | **3.62 (1.51-8.70) **** | 2.56 (.97-6.72) | **2.20 (1.34-3.61) **** |
| 6 | Search adjusted for each database | **2.01 (1.38-2.92) ***** | **1.42 (1.10-1.84) **** | **1.28 (1.08-1.52) **** |
| 7 | Multiple languages searched | **2.02 (1.39-2.94) ***** | **1.90 (1.48-2.44) ***** | **1.18 (1.00-1.39) *** |
| **Extended Methods** | |  |  |  |
| 8 | Grey literature searched | **2.10 (1.43-3.09) ***** | **1.32 (1.05-1.67) **** | 1.13 (.96-1.32) |
| 9 | Journals handsearched | 1.01 (.69-1.48) |  |  |
| 10 | Clinical trial registries searched | **2.76 (1.83-4.15) ***** | **1.55 (1.22-1.95)***** | **.**88 (.75-1.02) |
| 11 | Citation indices searched | 1.29 (.89-1.86) | 1.23 (.98-1.56) | 1.08 (.93-1.26) |
| 12 | References in articles reviewed | **2.24 (1.03-4.89) *** | 1.13 (.60-2.13) | 1.22 (.81-1.83) |
| 13 | Prominent authors contacted | 1.29 (.90-1.87) | **1.38 (1.09-1.74) **** | **1.19 (1.02-1.39) *** |
| **Search Process/Reporting** | |  |  |  |
| 14 | Search updated during process | 1.15 (.68-1.93) | .88 (.62-1.24) | **1.39 (1.11-1.74) **** |
| 15 | Search strategy peer-reviewed | 1.40 (.97-2.02) | 1.13 (.89-1.44) | 1.09 (.93-1.28) |
| 16 | SR registered in PROSPERO | **2.72 (1.87-3.96) ***** | 1.13 (.87-1.47) | **1.38 (1.16-1.65) ***** |
| 17 | Full strategy provided | **6.69 (1.57-28.43) **** | .90 (.60-1.37) | 1.29 (.97-1.71) |
| N=1271. Significant associations in bolded text. * p<.05, ** p<.01, ***p<.001. CI = confidence interval. SR = systematic review/meta-analysis. ‡ One point increase on five point scale. Odds ratios adjusted using the following covariates: librarian involvement, formal training in systematic review methodology, journal impact factor quartile, number of previous systematic reviews, use of a reporting guidelines, years of professional practice, time taken to write the review, year of publication and self-reported confidence in systematic review methodology and the topic area of the systematic review. | | | | |
